# Supplementary material for: Moderate heat stress prevented the observed biomass and yield stimulation caused by elevated CO2 in two well-watered wheat cultivars
Source: Plant Mol Biol. 2022 Jun 1;110(4-5):365–84. doi: 10.1007/s11103-022-01276-7 (PMC9646619; doi:10.1007/s11103-022-01276-7)
Supplement: Supplementary file 1 — Supplementary file1 (DOCX 1355 KB) [file 11103_2022_1276_MOESM1_ESM.docx]

**Table S1. Summary of statistics** **for gas exchange parameters** using anova test in R for effect of cultivar, elevated CO_2_ and heat stress (HS) on gas exchange parameters measured at 25°C leaf temperature at three-time points. T1 did not have HS treatment. T2 includes only HS1 at the vegetative stage, with two HS levels: Control and HS1. T3 included HS1 at the vegetative stage and HS2 at the reproductive stage with four HS levels: Control, HS1, HS2 and HS1+2. Both aCO_2_ and eCO_2_ grown plants were measured at 400 and 650 μl L^-1^. Growth CO_2_ measurements refer to measurement of ambient CO­_2_ grown plants at 400 (μl L^-1^) and elevated CO_2_ grown plants at (650 μl L^-1^). Significance levels are: *** = *p* < 0.001; ** = *p* < 0.01; * = *p* < 0.05; ns *p* > 1.

| Time Point | Parameter | Meas CO_2_ (μlL^-1^) | Main Effects | | | | | Interactions | | | |
| --- | --- | --- | --- | --- | --- | --- | --- | --- | --- | --- | --- |
|  |  |  | Cultivar | | CO_2_ | | HS | Cultivar*CO_2_ | CO_2_* HS | Cultivar*HS | Cultivar*CO_2_ * HS |
| Common and Growth CO_2_ | | | | | | | | | | | |
| T1 | **A_sat_**  (µmol m^-2^ s^-1^) | 400 | ns | | ** | |  | ns |  |  |  |
|  |  | 650 | ns | | ** | |  | * |  |  |  |
|  | **g_s_**  (mol m^-2^s^-1^) | 400 | ns | | *** | |  | ns |  |  |  |
|  |  | 650 | ns | | *** | |  | ns |  |  |  |
|  | **PWUE** (A_sat_/g_s_)  (µmol mol^-1^) | 400 | ns | | ** | |  | ns |  |  |  |
|  |  | 650 | ns | | ** | |  | ns |  |  |  |
|  | **Fv/Fm** | 400 | ns | | ns | |  | ns |  |  |  |
|  | **Fv'/Fm'** | 400 | *** | | ns | |  | ns |  |  |  |
|  | **Rd** (µmol m^-2^ s^-1^)  (µmol m^-2^s^-1^) | 400 | ns | | ns | |  | ns |  |  |  |
| T2 | **A_sat_**  (µmol m^-2^ s^-1^) | 400 | * | | ** | | ns | ns | ns | ns | ns |
|  |  | 650 | ** | | * | | ns | ns | ns | ns | ns |
|  | **g_s_**  (mol m^-2^s^-1^) | 400 | ns | | ns | | ns | ns | ns | ns | ns |
|  |  | 650 | ns | | ns | | ns | ns | ns | ns | ns |
|  | **PWUE** (A_sat_/g_s_)  (µmol mol^-1^) | 400 | ns | | ns | | ns | ns | ns | ns | ns |
|  |  | 650 | ** | | ns | | ns | ns | ns | ns | ns |
|  | **Fv/Fm** | 400 | ** | | ns | | ns | ns | ** | ns | ns |
|  | **Fv'/Fm'** | 400 | * | | * | | ns | ns | ns | ns | ns |
|  | **Rd** (µmol m^-2^ s^-1^)  (µmol m^-2^s^-1^) | 400 | *** | | ns | | ns | ns | ns | ns | ns |
| T3 | **A_sat_**  (µmol m^-2^ s^-1^) | 400 | * | | *** | | * | ns | ns | ns | ns |
|  |  | 650 | *** | | * | | ** | ns | ns | ns | ns |
|  | **g_s_**  (mol m^-2^s^-1^) | 400 | * | | *** | | *** | ns | ns | *** | ns |
|  |  | 650 | *** | | ** | | *** | ns | ns | *** | ns |
|  | **PWUE** (A_sat_/g_s_)  (µmol mol^-1^) | 400 | ns | | ns | | ** | * | ns | *** | ** |
|  |  | 650 | ns | | ns | | ** | ns | ns | *** | ns |
|  | **PNUE** (A_sat_/)  (µmol mol^-1^) | 400 |  | |  | |  |  |  |  |  |
|  | **Fv/Fm** | 400 | *** | | ns | | ns | *** | * | ns | * |
|  | **Fv'/Fm'** | 400 | ns | | ns | | ns | ns | ns | ns | ns |
|  | **Rd** (µmol m^-2^ s^-1^)  (µmol m^-2^s^-1^) | 400 | *** | | ns | | ns | * | ** | * | ns |
|  | **V_cmax_** | 400 | ns | | *** | | ns | ns | ns | ns | ns |
|  | **J_max_** | 400 | * | | ns | | ns | ns | ns | ns | ns |
|  | **V_cmax_ / J_max_** | 400 | ** | | *** | | ns | * | ns | ns | ns |
| Growth CO_2_ | | | | | | | | | | | |
| T3 | **A_sat_**  (µmol m^-2^ s^-1^) | Growth CO_2_ | ** | *** | | * | | ns | ns | ns | ns |
|  | **g_s_**  (mol m^-2^s^-1^) |  | ** | *** | | *** | | ns | ns | ** | ns |
|  | **PWUE** (A_sat_/g_s_)  (µmol mol^-1^) |  | ns | *** | | * | | ns | ns | *** | ** |
|  | **PNUE** (A_sat_/) |  | ns | ns | | ns | | ns | ns | ns | ns |
|  | **Rd** (µmol m^-2^ s^-1^)  **(µmol m^-2^s^-1^)** |  | **** | | *** | | ns | ns | ** | *** | ns |

Table S2. Response of Scout gas exchange parameters to elevated CO_2_ and heat stress. Summary of leaf gas exchange measured at two CO_2_ (400 and 650 μl L^-1^) partial pressures and 25^o^C leaf temperature for Scout grown at ambient CO_2_ (aCO_2_) or elevated CO_2_ (eCO_2_) and exposed to 1 and/or 2 heat stresses. Values are means ± SE (n= 9-10). Heat stress treatments include plants not exposed to any heat stress (control), plants exposed to heat stress 1 (HS1), heat stress 2 (HS2) and both heat stresses (HS1+2). Measurements for HS were performed at the recovery stage at respective time points.

| Scout  Parameters | Time  Point | Growth CO_2_ | Ambient | | | | Elevated | | | |
| --- | --- | --- | --- | --- | --- | --- | --- | --- | --- | --- |
|  |  | Meas CO_2_ / Heat Stress | Control | HS1 | HS2 | HS1+2 | Control | HS1 | HS2 | HS1+2 |
| A  (µmol m^-2^ s^-1^) | T1 | 400 | 24.6 ± 0.99 |  |  |  | 22.9 ± 0.49 |  |  |  |
|  |  | 650 | 31.4 ± 1.2 |  |  |  | 30.8 ± 0.67 |  |  |  |
|  | T2 | 400 | 24.4 ± 0.99 | 22.8 ± 0.84 |  |  | 21.6 ± 0.99 | 21.8 ± 1.02 |  |  |
|  |  | 650 | 32.4 ± 1.40 | 30.1 ± 1.51 |  |  | 29.2 ± 1.19 | 29 ± 1.09 |  |  |
|  | T3 | 400 | 17.6 ± 0.59 | 19.5 ± 0.64 | 18.6 ± 1.04 | 14.8 ± 1.12 | 17.1 ± 1.14 | 19.1 ± 0.96 | 14.8 ± 1.12 | 17 ± 1.29 |
|  |  | 650 | 22.5 ± 0.74 | 26.1 ± 0.97 | 25 ± 1.51 | 20.8 ± 1.47 | 22.8 ± 1.55 | 25.5 ± 1.47 | 20.8 ± 1.47 | 23.2 ± 1.9 |
| g_s_  (mol m^-2^s^-1^) | T1 | 400 | 0.35 ± 0.02 |  |  |  | 0.28 ± 0.01 |  |  |  |
|  |  | 650 | 0.36 ± 0.04 |  |  |  | 0.25 ± 0.01 |  |  |  |
|  | T2 | 400 | 0.36 ± 0.02 | 0.36 ± 0.02 |  |  | 0.33 ± 0.01 | 0.33 ± 0.02 |  |  |
|  |  | 650 | 0.31 ± 0.01 | 0.33 ± 0.01 |  |  | 0.33 ± 0.01 | 0.33 ± 0.01 |  |  |
|  | T3 | 400 | 0.29 ± 0.01 | 0.35 ± 0.01 | 0.31 ± 0.02 | 0.20 ± 0.02 | 0.23 ± 0.01 | 0.34 ± 0.02 | 0.20 ± 0.02 | 0.31 ± 0.02 |
|  |  | 650 | 0.26 ± 0.01 | 0.34 ± 0.01 | 0.27 ± 0.01 | 0.20 ± 0.02 | 0.23 ± 0.01 | 0.31 ± 0.02 | 0.20 ± 0.02 | 0.30 ± 0.02 |
| PWUE (A_sat_/g_s_)  (µmol mol^-1^) | T1 | 400 | 71.9 ± 3.3 |  |  |  | 83.2 ± 4.6 |  |  |  |
|  |  | 650 | 95.9 ± 10 |  |  |  | 123.5 ± 6.2 |  |  |  |
|  | T2 | 400 | 67.6 ± 3.7 | 65.2 ± 3.9 |  |  | 65.3 ± 2.2 | 66.6 ± 3.2 |  |  |
|  |  | 650 | 106 ± 9.5 | 90.6 ± 5.4 |  |  | 89.7 ± 4.0 | 86.9 ± 2.3 |  |  |
|  | T3 | 400 | 60.2 ± 2.3 | 56.0 ± 2.6 | 61.7 ± 3.6 | 81.0 ± 8.1 | 72.7 ± 3.4 | 56.6 ± 2.9 | 81.0 ± 8.1 | 55.3 ± 2.7 |
|  |  | 650 | 90.5 ± 6.3 | 77.4 ± 5.2 | 92.9 ± 4.3 | 125 ± 23.0 | 98.6 ± 4.2 | 82.4 ± 3.9 | 125 ± 23.0 | 75.3 ± 3.6 |
| Rd  (µmol m^-2^ s^-1^) | T1 | 400 | -1.11 ± 0.09 |  |  |  | -1.19 ± 0.08 |  |  |  |
|  | T2 | 400 | -0.54 ± 0.16 | -0.65 ± 0.16 |  |  | -0.76 ± 0.09 | -0.76 ± 0.14 |  |  |
|  | T3 | 400 | -1.04 ± 0.09 | -0.96 ± 0.08 | -1.08 ± 0.09 | -0.71 ± 0.15 | -1.06 ± 0.09 | -0.99 ± 0.16 | -0.71 ± 0.15 | -0.70 ± 0.05 |
| Fv/Fm | T1 | 400 | 0.81 ± 0.00 |  |  |  | 0.81 ± 0.00 |  |  |  |
|  | T2 | 400 | 0.81 ± 0.01 | 0.80 ± 0.01 |  |  | 0.80 ± 0.01 | 0.82 ± 0.00 |  |  |
|  | T3 | 400 | 0.79 ± 0.01 | 0.79 ± 0.01 | 0.79 ± 0.01 | 0.78 ± 0.01 | 0.76 ± 0.01 | 0.79 ± 0.01 | 0.78 ± 0.01 | 0.75 ± 0.02 |
| Fv'/Fm' | T1 | 400 | 0.48 ± 0.01 |  |  |  | 0.49 ± 0.01 |  |  |  |
|  | T2 | 400 | 0.48 ± 0.01 | 0.48 ± 0.00 |  | 0.45 ± 0.02 | 0.50 ± 0.01 | 0.50 ± 0.00 |  |  |
|  | T3 | 400 | 0.50 ± 0.01 | 0.50 ± 0.01 | 0.49 ± 0.01 | 0.52 ± 0.01 | 0.52 ± 0.02 | 0.50 ± 0.01 | 0.45 ± 0.02 | 0.48 ± 0.02 |
| PNUE | T3 | Growth CO_2_ | 317 ± 52 |  |  | 270 ± 32 | 247 ± 25 |  |  | 280 ± 50 |
| V_cmax_ | T3 | NA | 84.2 ± 2.7 |  |  | 82.2 ± 5.3 | 73.9 ± 3.6 |  |  | 72.8 ± 5.6 |
| J_max_ | T3 | NA | 134 ± 5 |  |  | 137 ± 9 | 133 ± 7 |  |  | 138 ± 8 |
| V_cmax_/ J_max_ | T3 | NA | 0.63 ± 0.01 |  |  | 0.60 ± 0.01 | 0.55 ± 0.01 |  |  | 0.52 ± 0.02 |

Table S3. Response of Yitpi gas exchange parameters to growth at elevated CO_2_ and heat stress. Summary of leaf gas exchange measured at two CO_2_ (400 and 650 μl L^-1^) partial pressures and 25^o^C leaf temperature for Yitpi grown at ambient CO_2_ (aCO_2_) or elevated CO_2_ (eCO_2_) and exposed to 1 and/or 2 heat stresses. Values are means ± SE (n= 9-10). Heat stress treatments include plants not exposed to any heat stress (control), plants exposed to heat stress 1 (HS1), heat stress 2 (HS2) and both heat stresses (HS1+2). Measurements for HS were performed at the recovery stage at respective time points.

| Yitpi  Parameters | Time Point | Growth CO2 | Ambient | | | | Elevated | | | |
| --- | --- | --- | --- | --- | --- | --- | --- | --- | --- | --- |
|  |  | **Meas CO_2 /_ Heat Stress** | **Control** | **HS1** | **HS2** | **HS1+2** | **Control** | **HS1** | **HS2** | **HS1+2** |
| A  (µmol m^-2^ s^-1^) | T1 | 400 | 24.8 ± 0.77 |  |  |  | 21 ± 0.63 |  |  |  |
|  |  | 650 | 33.9 ± 0.93 |  |  |  | 29.5 ± 0.57 |  |  |  |
|  | T2 | 400 | 26 ± 0.63 | 24.2 ± 1.01 |  |  | 23.4 ± 1.12 | 22.9 ± 1.49 |  |  |
|  |  | 650 | 36.2 ± 0.84 | 34 ± 1.30 |  |  | 31.9 ± 1.99 | 32.5 ± 2.18 |  |  |
|  | T3 | 400 | 20 ± 1.09 | 20.9 ± 1.13 | 20.1 ± 0.69 | 18.3 ± 0.81 | 16.3 ± 0.91 | 20.2 ± 1.20 | 18.3 ± 0.81 | 18.4 ± 0.81 |
|  |  | 650 | 27.6 ± 1.64 | 29.2 ± 1.46 | 27.7 ± 0.92 | 25.9 ± 1.36 | 23.3 ± 1.22 | 28.6 ± 1.49 | 25.9 ± 1.36 | 26.7 ± 1.14 |
| g_s_  (mol m^-2^s^-1^) | T1 | 400 | 0.35 ± 0.02 |  |  |  | 0.25 ± 0.01 |  |  |  |
|  |  | 650 | 0.37 ± 0.02 |  |  |  | 0.27 ± 0.01 |  |  |  |
|  | T2 | 400 | 0.38 ± 0.02 | 0.33 ± 0.02 |  |  | 0.34 ± 0.01 | 0.31 ± 0.01 |  |  |
|  |  | 650 | 0.35 ± 0.01 | 0.31 ± 0.01 |  |  | 0.29 ± 0.01 | 0.30 ± 0.02 |  |  |
|  | T3 | 400 | 0.30 ± 0.02 | 0.34 ± 0.02 | 0.37 ±0.01 | 0.35 ± 0.01 | 0.27 ± 0.01 | 0.33 ± 0.02 | 0.35 ± 0.01 | 0.29 ± 0.01 |
|  |  | 650 | 0.29 ± 0.02 | 0.34 ± 0.01 | 0.38 ±0.01 | 0.35 ± 0.01 | 0.27 ± 0.01 | 0.32 ± 0.02 | 0.35 ± 0.01 | 0.30 ± 0.01 |
| PWUE (A_sat_/g_s_)  (µmol mol^-1^) | T1 | 400 | 72.4 ± 3.8 |  |  |  | 84.1 ± 5.1 |  |  |  |
|  |  | 650 | 93.1 ± 5.6 |  |  |  | 110 ± 3.6 |  |  |  |
|  | T2 | 400 | 68.3 ± 3.0 | 75.7 ± 4.4 |  |  | 68.3 ± 3.5 | 71.9 ± 3.9 |  |  |
|  |  | 650 | 103 ± 5.9 | 110 ± 6.1 |  |  | 108 ± 7.0 | 109 ± 6.3 |  |  |
|  | T3 | 400 | 66.1 ± 2.3 | 60.5 ± 2.2 | 54.8 ± 1.9 | 52.0 ± 1.8 | 61.1 ± 2.3 | 61.1 ± 2.5 | 52.0 ± 1.8 | 62.7 ± 2.9 |
|  |  | 650 | 94.1 ± 4.6 | 85.9 ± 2.6 | 73.3 ± 2.7 | 73.8 ± 3.3 | 88.7 ± 6.1 | 89.7 ± 4.2 | 73.8 ± 3.3 | 88.5 ± 2.9 |
| Rd  (µmol m^-2^ s^-1^) | T1 | 400 | -1.15 ± 0.59 |  |  |  | -1.19 ± 0.10 |  |  |  |
|  | T2 | 400 | -0.69 ± 0.10 | -1.24 ± 0.14 |  |  | -1.06 ± 0.12 | -1.06 ± 0.08 |  |  |
|  | T3 | 400 | -0.23 ± 0.28 | -0.30 ± 0.15 | -1.16 ± 0.13 | -0.61 ± 0.06 | -0.63 ± 0.09 | -0.70 ± 0.05 | -0.61 ± 0.06 | -0.83 ± 0.24 |
| Fv/Fm | T1 | 400 | 0.81 ± 0.00 |  |  |  | 0.81 ± 0.00 |  |  |  |
|  | T2 | 400 | 0.82 ± 0.00 | 0.81 ± 0.00 |  |  | 0.81 ± 0.00 | 0.82 ± 0.00 |  |  |
|  | T3 | 400 | 0.81 ± 0.00 | 0.77 ± 0.01 | 0.81 ± 0.00 | 0.81 ± 0.01 | 0.80 ± 0.00 | 0.81 ± 0.00 | 0.81 ± 0.01 | 0.81 ± 0.00 |
| Fv'/Fm' | T1 | 400 | 0.46 ± 0.00 |  |  |  | 0.45 ± 0.00 |  |  |  |
|  | T2 | 400 | 0.48 ± 0.00 | 0.47 ± 0.01 |  | 0.50 ± 0.01 | 0.48 ± 0.01 | 0.48 ± 0.01 |  |  |
|  | T3 | 400 | 0.50 ± 0.01 | 0.50 ± 0.01 | 0.51 ± 0.00 | 0.51 ± 0.01 | 0.50 ± 0.01 | 0.48 ± 0.01 | 0.50 ± 0.01 | 0.49 ± 0.01 |
| PNUE | T3 | Growth CO_2_ | 283 ± 16 |  |  | 284 ± 15 | 267 ± 26 |  |  | 280 ± 14 |
| V_cmax_ | T3 | NA | 81.5 ± 2.9 |  |  | 90.3 ± 4.2 | 70.8 ± 4.9 |  |  | 80.1 ± 3.4 |
| J_max_ | T3 | NA | 144 ± 6 |  |  | 161 ± 10 | 134 ± 9 |  |  | 148 ± 5 |
| V_cmax_/ J_max_ | T3 | NA | 0.56 ± 0.01 |  |  | 0.56 ± 0.01 | 0.52 ± 0.01 |  |  | 0.54 ± 0.01 |

Table S4. Summary of statistics for biomass parameters using anova test in R for effect of cultivar, elevated CO_2_ and heat stress (HS) on plant dry mass (DM) and morphological parameters measured at four-time points. Significance levels are: *** = *p* < 0.001; ** = *p* < 0.01; * = *p* < 0.05; ns *p* > 1.

| Time Point | Parameter  (Mean plant^-1^) | Main Effects | | | | | Interactions | | | | | |
| --- | --- | --- | --- | --- | --- | --- | --- | --- | --- | --- | --- | --- |
|  |  | Cultivar | CO_2_ | | HS | | Cultivar × CO_2_ | CO_2_ × HS | | Cultivar × HS | | Cultivar × CO_2_ × HS |
| T1 | Leaf number (n) | *** | | ns | |  | ns |  |  | |  | |
|  | Leaf area (cm^2^) | ns | | ns | |  | ns |  |  | |  | |
|  | Leaf DM (g) | * | | * | |  | ns |  |  | |  | |
|  | Stem DM (g) | ** | | ns | |  | ns |  |  | |  | |
|  | Root DM (g) | ns | | ns | |  | ns |  |  | |  | |
|  | Shoot DM (g) | ** | | * | |  | ns |  |  | |  | |
|  | Total DM (g) | ns | | ns | |  | ns |  |  | |  | |
| T2 | Height (cm) | *** | | ns | | ns | ns | ns | ** | | ns | |
|  | Tiller number | *** | | ns | | ns | ns | ns | ns | | ns | |
|  | Leaf number | *** | | ns | | ns | ns | ns | ns | | ns | |
|  | Leaf area (cm^2^) | *** | | ns | | ns | ns | ns | ns | | ns | |
|  | Leaf DM (g) | *** | | ns | | ns | ns | ns | ns | | ns | |
|  | Stem DM (g) | ns | | ** | | ns | ns | ns | ns | | ns | |
|  | Root DM (g) | *** | | ns | | ns | ns | ns | ns | | ns | |
|  | Shoot DM (g) | *** | | ** | | ns | ns | ns | ns | | ns | |
|  | Total DM (g) | *** | | * | | ns | ns | ns | ns | | ns | |
|  | Height (cm) | *** | | *** | | ns | ns | ns | ** | | ns | |
| T3 | Tiller number | *** | | ns | | ns | * | ns | ns | | ns | |
|  | Leaf number | *** | | * | | ns | ns | ns | ns | | ns | |
|  | Leaf area (cm^2^) | *** | | ns | | ns | ns | ns | ns | | ns | |
|  | Leaf DM (g) | *** | | * | | ns | ns | ns | ns | | ns | |
|  | Stem DM (g) | *** | | *** | | ns | ns | ns | ns | | ns | |
|  | Root DM (g) | *** | | * | | ns | ns | ns | ns | | ns | |
|  | Shoot DM (g) | *** | | *** | | ns | ns | ns | ns | | ns | |
|  | Total DM (g) | *** | | *** | | ns | ns | ns | ns | | ns | |
|  | Total N uptake (g plant^-1^) | *** | | ns | | ** | ns | ns | ns | | ns | |
| T4 | Tiller number | *** | | ns | | ns | * | ns | ns | | ns | |
|  | Ear no/tiller no (ratio) | ** | | ns | | ns | ns | ns | ns | | ns | |
|  | Root DM (g) | *** | | ns | | ns | ns | ns | ns | | ns | |
|  | Shoot DM (g) | *** | | * | | ns | ns | ** | ns | | ns | |
|  | Total DM (g) | *** | | * | | ns | ns | ** | ns | | ns | |
|  | Grain yield (g) | ns | | ** | | ns | ns | ** | ns | | ns | |
|  | Grain number | *** | | ns | | ns | ns | ns | ** | | ns | |
|  | Grain size (mg grain^-1^) | *** | | ** | | ns | ** | ns | ns | | *** | |
|  | Harvest index | *** | | * | | ns | * | ns | ns | | ns | |
|  | Grain protein (%) | *** | | * | | ns | ** | * | ns | | * | |
|  | Total N uptake (g plant^-1^) | *** | | ns | | ns | ns | *** | ns | | ns | |
|  | N utilization efficiency  (g yield (g N uptake)^-1^) | *** | | ns | | ns | ns | ns | ns | | ns | |

Table S5. Response of plant morphological parameters, dry mass and yield to elevated CO_2_ and HS. Summary of plant dry mass (DM) and morphological parameters measured at different time points for Scout and Yitpi grown at ambient CO_2_ (aCO_2_) or elevated CO_2_ (eCO_2_) and exposed to 1 and /or 2 heat stresses. Values are means ± SE (n= 9-10). Heat stress levels include plants not exposed to any heat stress (control), plants exposed to heat stress 1 (HS1), heat stress 2 (HS2) and both heat stresses (HS1+2).

| Parameter | Time Point | CO_2_ / Heat Stress | Scout | | | | Yitpi | | | |
| --- | --- | --- | --- | --- | --- | --- | --- | --- | --- | --- |
|  |  |  | **Control** | **HS1** | **HS2** | **HS1+2** | **Control** | **HS1** | **HS2** | **HS1+2** |
| Height  (cm plant^-1^) | T2 | aCO_2_ | 74 ± 1 | 69 ± 1 |  |  | 42 ± 3 | 52 ± 2 |  |  |
|  |  | eCO_2_ | 73 ± 1 | 68 ± 1 |  |  | 50 ± 3 | 52 ± 3 |  |  |
|  | T3 | aCO_2_ | 74 ± 2 | 71 ± 1 | 73 ± 1 | 73 ± 1 | 75 ± 2 | 82 ± 1 | 81 ± 1 | 76 ± 1 |
|  |  | eCO_2_ | 79 ± 1 | 76 ± 1 | 76 ± 1 | 76 ± 2 | 86 ± 1 | 85 ± 2 | 87 ± 1 | 81 ± 1 |
| Leaf DM  (g plant^-1^) | T1 | aCO_2_ | 0.21 ± 0.03 |  |  |  | 0.34 ± 0.04 |  |  |  |
|  |  | eCO_2_ | 0.34 ± 0.03 |  |  |  | 0.40 ± 0.04 |  |  |  |
|  | T2 | aCO_2_ | 1.6 ± 0.2 | 1.3 ± 0.2 |  |  | 4.3 ± 0.2 | 4.3 ± 0.4 |  |  |
|  |  | eCO_2_ | 1.6 ± 0.1 | 1.7 ± 0.1 |  |  | 4.5 ± 0.1 | 4.7 ± 0.2 |  |  |
|  | T3 | aCO_2_ | 1.4 ± 0.2 | 1.1 ± 0.1 | 1.2 ± 0.2 | 1.5 ± 0.2 | 4.3 ± 0.3 | 4.8 ± 0.3 | 3.9 ± 0.4 | 4.4 ± 0.3 |
|  |  | eCO_2_ | 1.5 ± 0.1 | 2.0 ± 0.2 | 1.6 ± 0.2 | 1.8 ± 0.2 | 4.7 ± 0.5 | 4.7 ± 0.4 | 5.4 ± 0.3 | 4.9 ± 0.2 |
| Stem DM  (g plant^-1^) | T1 | aCO_2_ | 0.21 ± 0.03 |  |  |  | 0.34 ± 0.04 |  |  |  |
|  |  | eCO_2_ | 0.13 ± 0.02 |  |  |  | 0.18 ± 0.01 |  |  |  |
|  | T2 | aCO_2_ | 7.2 ± 0.6 | 5.8 ± 0.5 |  |  | 5.8 ± 0.5 | 6.6 ± 0.7 |  |  |
|  |  | eCO_2_ | 8.0 ± 0.4 | 7.1 ± 0.2 |  |  | 8.0 ± 0.4 | 7.4 ± 0.7 |  |  |
|  | T3 | aCO_2_ | 14.5 ± 1.5 | 10.6 ± 0.9 | 12.2 ± 1.8 | 13.9 ± 1.6 | 17.8 ± 1.2 | 19.0 ± 1.1 | 18.9 ± 1.2 | 16.7 ± 1.3 |
|  |  | eCO_2_ | 16.0 ± 1.1 | 18.1 ± 1.1 | 15.6 ± 1.6 | 17.3 ± 1.7 | 24.3 ± 1.6 | 23.7 ± 1.2 | 24.1 ± 0.7 | 21.1 ± 1.4 |
| Shoot DM  (g plant^-1^) | T1 | aCO_2_ | 0.31 ± 0.03 |  |  |  | 0.50 ± 0.05 |  |  |  |
|  |  | eCO_2_ | 0.48 ± 0.04 |  |  |  | 0.59 ± 0.06 |  |  |  |
|  | T2 | aCO_2_ | 8.8 ± 0.9 | 7.1 ± 0.7 |  |  | 10.2 ± 0.7 | 11.0 ± 0.8 |  |  |
|  |  | eCO_2_ | 9.7 ± 0.4 | 8.8 ± 0.3 |  |  | 12.5 ± 0.9 | 12.1 ± 0.9 |  |  |
|  | T3 | aCO_2_ | 15.9 ± 1.7 | 11.7 ± 1.0 | 13.4 ± 2.0 | 15.4 ± 1.9 | 22.2 ± 1.2 | 21.5 ± 2.5 | 22.8 ± 1.1 | 21.1 ± 1.0 |
|  |  | eCO_2_ | 17.6 ± 1.2 | 20.1 ± 1.3 | 17.2 ± 1. | 19.1 ± 1.9 | 29.0 ± 1.9 | 28.5 ± 1.2 | 29.5 ± 0.7 | 26.1 ± 1.5 |
|  | T4 | aCO_2_ | 14.5 ± 1.1 |  | 21.8 ± 3.4 | 18.7 ± 2.6 | 28.8 ± 2.9 |  | 37.6 ± 2.8 | 34.2 ± 1.5 |
|  |  | eCO_2_ | 24.3 ± 0.8 |  | 22.1 ± 1.1 | 16.7 ± 0.7 | 37.1 ± 2.3 |  | 42.8 ± 2.0 | 34.7 ± 3.2 |
| Root DM  (g plant^-1^) | T1 | aCO_2_ | 0.5 ± 0.1 |  |  |  | 0.6 ± 0.1 |  |  |  |
|  |  | eCO_2_ | 0.6 ± 0.1 |  |  |  | 0.7 ± 0.1 |  |  |  |
|  | T2 | aCO_2_ | 1.1 ± 0.23 | 0.7 ± 0.12 |  |  | 2.5 ± 0.37 | 2.5 ± 0.19 |  |  |
|  |  | eCO_2_ | 0.9 ± 0.09 | 0.9 ± 0.06 |  |  | 2.2 ± 0.24 | 2.7 ± 0.39 |  |  |
|  | T3 | aCO_2_ | 0.8 ± 0.18 | 0.5 ± 0.07 | 0.6 ± 0.16 | 0.6 ± 0.14 | 1.7 ± 0.13 | 2.1 ± 0.32 | 1.5 ± 0.13 | 1.9 ± 0.24 |
|  |  | eCO_2_ | 0.7 ± 0.09 | 0.9 ± 0.13 | 0.8 ± 0.22 | 0.8 ± 0.16 | 1.8 ± 0.12 | 2.6 ± 0.39 | 2.1 ± 0.23 | 2.8 ± 0.44 |
|  | T4 | aCO_2_ | 0.3 ± 0.07 |  | 0.4 ± 0.08 | 0.5 ± 0.13 | 1 ± 0.32 |  | 1.0 ± 0.17 | 1.4 ± 0.28 |
|  |  | eCO_2_ | 0.4 ± 0.09 |  | 0.4 ± 0.10 | 0.3 ± 0.12 | 1.0 ± 0.19 |  | 1.2 ± 0.29 | 1.3 ± 0.29 |
| Leaf Area  (cm^2^ plant^-1^) | T1 | aCO_2_ | 9.8± 2.4 |  |  |  | 24.9 ± 6.6 |  |  |  |
|  |  | eCO_2_ | 18.6 ± 3.8 |  |  |  | 24.9 ± 6.5 |  |  |  |
|  | T2 | aCO_2_ | 291 ± 51 | 159 ± 24 |  |  | 630 ± 96 | 560 ± 18 |  |  |
|  |  | eCO_2_ | 237 ± 28 | 204 ± 20 |  |  | 693 ± 81 | 648 ± 25 |  |  |
|  | T3 | aCO_2_ | 231 ± 39 | 170 ± 29 | 184 ± 47 | 240 ± 51 | 820 ± 89 | 981 ± 98 | 754 ± 114 | 902 ± 89 |
|  |  | eCO_2_ | 258 ± 26 | 321 ± 44 | 248 ± 52 | 287 ± 45 | 754 ± 63 | 870 ± 96 | 1015 ± 51 | 910 ± 50 |
| Leaf Mass Area  (g m^-2^) | T1 | aCO_2_ | 333 ± 98 |  |  |  | 239 ± 62 |  |  |  |
|  |  | eCO_2_ | 316 ± 87 |  |  |  | 269 ± 56 |  |  |  |
|  | T2 | aCO_2_ | 47 ± 12 | 130 ± 61 |  |  | 81 ± 1 | 79 ± 9 |  |  |
|  |  | eCO_2_ | 75 ± 7 | 96 ± 17 |  |  | 73 ± 7 | 72 ± 3 |  |  |
|  | T3 | aCO_2_ | 64 ± 2 | 68 ± 3 | 76 ± 06 | 79 ± 10 | 54 ± 2 | 51 ± 2 | 54 ± 2 | 50 ± 1 |
|  |  | eCO2 | 62 ± 2 | 64 ± 2 | 75 ± 11 | 69 ± 03 | 62 ± 0.3 | 56 ± 3 | 53 ± 1 | 55 ± 1 |
| Leaf Size  (cm^2^ plant^-1^) | T1 | aCO_2_ | 0.9 ± 0.2 |  |  |  | 1.5 ± 0.3 |  |  |  |
|  |  | eCO_2_ | 1.6 ± 0.3 |  |  |  | 1.6 ± 0.3 |  |  |  |
|  | T2 | aCO_2_ | 9.2 ± 1.3 | 7.0 ± 1.0 |  |  | 15.0 ± 2.0 | 12.3 ± 0.7 |  |  |
|  |  | eCO_2_ | 8.1 ± 0.8 | 7.2 ± 0.8 |  |  | 14.7 ± 1.7 | 14.0 ± 0.8 |  |  |
|  | T3 | aCO_2_ | 9.4 ± 0.9 | 7.6 ± 0.6 | 8.1 ± 1.3 | 8.2 ± 1.1 | 15.2 ± 0.6 | 16.0 ± 1.2 | 13.9 ± 1.0 | 16.4 ± 1.2 |
|  |  | eCO_2_ | 10.2 ± 1.0 | 8.3 ± 0.5 | 9.5 ± 1.2 | 8. ± 1.0 | 14.1 ± 1.1 | 15.3 ± 0.8 | 16.3 ± 0.7 | 16.2 ± 1.3 |
| Leaf Number  (plant^-1^) | T1 | aCO_2_ | 10.4 ± 0.9 |  |  |  | 15.9 ± 0.5 |  |  |  |
|  |  | eCO_2_ | 12.1 ± 0.9 |  |  |  | 14.7 ± 0.9 |  |  |  |
|  | T2 | aCO_2_ | 28.8 ± 3.5 | 25.8 ± 3.4 |  |  | 43.6 ± 2.0 | 46.3 ± 2.4 |  |  |
|  |  | eCO_2_ | 29.5 ± 1.7 | 29.3 ± 2.4 |  |  | 47.2 ± 2.1 | 47.6 ± 3.1 |  |  |
|  | T3 | aCO_2_ | 23.5 ± 2.1 | 21.3 ± 1.9 | 20.2 ± 1.7 | 26.0 ± 2.9 | 53.7 ± 5.5 | 60.8 ± 3.6 | 53.6 ± 6.5 | 54.4 ± 3.0 |
|  |  | eCO_2_ | 25.8 ± 1.6 | 37.4 ± 3.43 | 24.1 ± 2.6 | 31.8 ± 2.4 | 55.8 ± 5.0 | 57.5 ± 5.9 | 62.5 ± 3.1 | 58.1 ± 3.9 |
| Tiller Number  (plant^-1^) | T2 | aCO_2_ | 6.7 ± 0.7 | 6.8 ± 0.7 |  |  | 10.8 ± 0.6 | 11.0 ± 0.6 |  |  |
|  |  | eCO_2_ | 7.1 ± 0.4 | 8.1 ± 0.3 |  |  | 11.3 ± 0.6 | 11.5 ± 0.4 |  |  |
|  | T3 | aCO_2_ | 8.2 ± 0.3 | 6.9 ± 0.5 | 7.2 ± 0.5 | 7.7 ± 1.1 | 18.9 ± 1.7 | 20.2 ± 0.9 | 20.1 ± 1.5 | 19.3 ± 1.3 |
|  |  | eCO_2_ | 8.0 ± 0.5 | 11.5 ± 1.0 | 7.5 ± 0.5 | 10.5 ± 0.8 | 18.8 ± 1.7 | 19.6 ± 1.0 | 20.0 ± 1.0 | 18.8 ± 1.2 |
|  | T4 | aCO_2_ | 7.2 ± 0.4 |  | 8.8 ± 0.8 | 8.4 ± 0.5 | 16.8 ± 1.8 |  | 16.2 ± 1.1 | 18.2 ± 2.4 |
|  |  | eCO_2_ | 8.6 ± 0.9 |  | 8.4 ± 0.6 | 9.2 ± 0.3 | 15.8 ± 0.9 |  | 17.6 ± 0.7 | 13.6 ± 0.9 |
| Ear Number  (plant^-1^) | T2 | aCO_2_ | 3.2 ± 0.2 | 3.7 ± 0.2 |  |  | 0.6 ± 0.1 | 1.7 ± 0.3 |  |  |
|  |  | eCO_2_ | 3 ± 0.1 | 3.5 ± 0.2 |  |  | 1.7 ± 0.3 | 2 ± 0.2 |  |  |
|  | T3 | aCO_2_ | 8.2 ± 0.3 | 6.9 ± 0.5 | 7.2 ± 0.5 | 8.5 ± 0.8 | 10.2 ± 0.7 | 10.3 ± 0.4 | 11.6 ± 0.7 | 10.9 ± 0.7 |
|  |  | eCO_2_ | 7.9 ± 0.5 | 11.5 ± 1.0 | 8 ± 0.6 | 10.3 ± 0.8 | 12.8 ± 0.6 | 12.3 ± 0.9 | 12.5 ± 0.5 | 11.4 ± 0.7 |
|  | T4 | aCO_2_ | 7.7 ± 0.2 |  | 8.8 ± 0.8 | 8.4 ± 0.6 | 14.8 ± 1.2 |  | 15.8 ± 1 | 15.2 ± 1 |
|  |  | eCO_2_ | 9.2 ± 0.8 |  | 9 ± 0.7 | 9 ± 0.3 | 15 ± 1 |  | 16.6 ± 1.1 | 13.4 ± 1 |
| Ear No /  Tiller No  (ratio) | T2 | aCO_2_ | 0.50 ± 0.04 | 0.59 ± 0.05 |  |  | 0.07 ± 0.02 | 0.16 ± 0.03 |  |  |
|  |  | eCO_2_ | 0.44 ± 0.04 | 0.43 ± 0.02 |  |  | 0.16 ± 0.04 | 0.17 ± 0.03 |  |  |
|  | T3 | aCO_2_ | 1 ± 0 | 1 ± 0 | 1 ± 0 | 1 ± 0 | 0.58 ± 0.06 | 0.52 ± 0.03 | 0.56 ± 0.04 | 0.58 ± 0.05 |
|  |  | eCO_2_ | 0.99 ± 0.01 | 1 ± 0 | 1 ± 0 | 0.98 ± 0.02 | 0.72 ± 0.05 | 0.63 ± 0.04 | 0.63 ± 0.03 | 0.62 ± 0.04 |
|  | T4 | aCO_2_ | 1 ± 0 |  | 1 ± 0 | 1 ± 0.04 | 0.89 ± 0.05 |  | 0.98 ± 0.01 | 0.86 ± 0.06 |
|  |  | eCO_2_ | 1 ± 0 |  | 1 ± 0.04 | 0.98 ± 0.02 | 0.95 ± 0.03 |  | 0.94 ± 0.03 | 0.98 ± 0.02 |
|  | T0 | aCO_2_ | 0.04 ± 0.001 |  |  |  | 0.04 ± 0.003 |  |  |  |
|  | T1 | aCO_2_ | 0.82 ± 0.18 |  |  |  | 1.16 ± 0.15 |  |  |  |
| Total Plant DM  (g plant^-1^) |  | eCO_2_ | 1.17 ± 0.17 |  |  |  | 1.32 ± 0.15 |  |  |  |
|  | T2 | aCO_2_ | 9.9 ± 1.0 | 7.8 ± 0.8 |  |  | 12.8 ± 0.8 | 13.6 ± 0.9 |  |  |
|  |  | eCO_2_ | 10.6 ± 0.5 | 9.7 ± 0.3 |  |  | 14.8 ± 1.1 | 14.9 ± 1.3 |  |  |
|  | T3 | aCO_2_ | 16.8 ± 1.8 | 12.2 ± 1.1 | 14.0 ± 2.2 | 16.1 ± 2.0 | 23.9 ± 1.1 | 26.1 ± 0.7 | 24.4 ± 1.2 | 23.1 ± 1.0 |
|  |  | eCO_2_ | 18.3 ± 1.3 | 21.0 ± 1.4 | 18.1 ± 2.0 | 20.0 ± 2.1 | 30.8 ± 1.9 | 31.1 ± 1.2 | 31.6 ± 0.7 | 28.9 ± 1.8 |
|  | T4 | aCO_2_ | 14.9 ± 1.8 |  | 22.3 ± 3.4 | 19.2 ± 2.7 | 29.8 ± 3.1 |  | 38.6 ± 2.9 | 35.7 ± 1.7 |
|  |  | eCO_2_ | 24.9 ± 0.8 |  | 20.0 ± 1.1 | 17.1 ± 0.6 | 38.1 ± 2.5 |  | 44.1 ± 2.3 | 36.0 ± 3.4 |
| Grains Per Ear  (plant^-1^) | T4 | aCO_2_ | 29 ± 2 |  | 31 ± 3 | 29 ± 3 | 22 ± 2 |  | 30 ± 2 | 27 ± 2 |
|  |  | eCO_2_ | 36 ± 4 |  | 32 ± 1 | 26 ± 1 | 29 ± 1 |  | 28 ± 2 | 27 ± 2 |
| Total Grain Number (plant^-1^) | T4 | aCO_2_ | 230 ± 15 |  | 273 ± 34 | 247 ± 36 | 328 ± 32 |  | 471 ± 41 | 405 ± 7 |
|  |  | eCO_2_ | 326 ± 11 |  | 287 ± 19 | 237 ± 8 | 433 ± 37 |  | 458 ± 27 | 364 ± 38 |
| Mean Grain Size  (mg grain^-1^) | T4 | aCO_2_ | 37 ± 1 |  | 38 ± 1 | 43 ± 1 | 28 ± 1 |  | 28 ± 1 | 27 ± 1 |
|  |  | eCO_2_ | 42 ± 2 |  | 40 ± 2 | 38 ± 1 | 32 ± 1 |  | 32 ± 1 | 35 ± 2 |
| Grain yield  (g plant^-1^) | T4 | aCO_2_ | 8.5 ± 0.6 |  | 10.9 ± 2.0 | 10.7 ± 1.7 | 9.1 ± 1.0 |  | 13.2 ± 1.0 | 11.1 ± 0.4 |
|  |  | eCO_2_ | 14.0 ± 0.6 |  | 11.6 ± 0.9 | 9.0 ± 0.5 | 13.7 ± 1.0 |  | 14.8 ± 0.8 | 12.8 ± 1.3 |
| Harvest Index | T4 | aCO_2_ | 0.58 ±0.01 |  | 0.49 ±0.03 | 0.56 ±0.01 | 0.31 ±0.01 |  | 0.35 ±0.01 | 0.32 ±0.01 |
|  |  | eCO_2_ | 0.57 ±0.01 |  | 0.52 ±0.01 | 0.53 ±0.01 | 0.36 ±0.01 |  | 0.35 ±0.03 | 0.37 ±0.02 |
| Total N uptake  (g N plant^-1^) | T3 | aCO_2_ | 0.21 ± 0.02 |  |  | 0.19 ± 0.04 | 0.41 ± 0.06 |  |  | 0.53 ± 0.04 |
|  |  | eCO_2_ | 0.21 ± 0.02 |  |  | 0.32 ± 0.06 | 0.37 ± 0.02 |  |  | 0.57 ± 0.06 |
|  | T4 | aCO_2_ | 0.31 ± 0.02 |  |  | 0.47 ± 0.04 | 0. 50 ± 0.04 |  |  | 0.70 ± 0.1 |
|  |  | eCO_2_ | 0.54 ± 0.03 |  |  | 0.31 ± 0.01 | 0.77 ± 0.06 |  |  | 0.60 ± 0.07 |
| Grain Protein  (%) | T4 | aCO_2_ | 18 ± 0.6 |  |  | 18.1 ± 0.1 | 22.5 ± 0.2 |  |  | 20.3 ± 0.6 |
|  |  | eCO_2_ | 18 ± 1 |  |  | 18 ± 0.6 | 18.7 ± 0.3 |  |  | 20.1 ± 0.8 |
| N utilization efficiency  (g yield (g N uptake) ^-1^) | T4 | aCO_2_ | 28.1 ± 1.1 |  |  | 25.7 ± 2 | 18.1 ± 0.8 |  |  | 16.9 ± 2 |
|  |  | eCO_2_ | 26.0 ± 1 |  |  | 26.6 ± 1.2 | 17.9 ± 0.2 |  |  | 21.8 ± 2.1 |

Table S6. Summary of plant nitrogen content parameters. Summary of nitrogen content determined from flag leaf measured for gas exchange from Scout and Yitpi grown at ambient or elevated CO_2_ and exposed to 1 and /or 2 heat stresses (HS). Values are means ± SE (n= 9-10). Heat stress levels include plants not exposed to any heat stress (control), plants exposed to only HS1, HS2 or both HS (HS1+2).

| Parameter | Time Point | Cultivar | Scout | | | | Yitpi | | | |
| --- | --- | --- | --- | --- | --- | --- | --- | --- | --- | --- |
|  |  | **Heat Stress** | **Control** | **HS1** | **HS2** | **HS1+2** | **Control** | **HS1** | **HS2** | **HS1+2** |
|  |  | **CO_2_** |  |  |  |  |  |  |  |  |
| Grain N (mg g^-1^) | T4 | aCO_2_ | 31 ± 1 |  |  | 31 ± 0 | 39 ± 0 |  |  | 35 ± 1 |
|  |  | eCO_2_ | 31 ± 1 |  |  | 32 ± 0 | 32 ± 0 |  |  | 35 ± 1 |
| Flag Leaf LMA  (g m^-2^) | T2 | aCO_2_ | 32 ± 6 | 40 ± 1 |  |  | 42 ± 2 | 43 ± 3 |  |  |
|  |  | eCO_2_ | 42 ± 2 | 41 ± 1 |  |  | 42 ± 2 | 42 ± 2 |  |  |
|  | T3 | aCO_2_ | 33 ± 1 | 47 ± 4 | 37 ± 4 | 35 ± 3 | 34 ± 1 | 39 ± 9 | 29 ± 6 | 37 ± 2 |
|  |  | eCO_2_ | 32 ± 1 | 37 ± 2 | 36 ± 3 | 32 ± 4 | 34 ± 1 | 38 ± 2 | 37 ± 1 | 37 ± 2 |
| Flag Leaf N  (mg g^-1^) | T2 | aCO_2_ | 42 ± 1 | 37 ± 1 |  |  | 41 ± 2 | 37 ± 2 |  |  |
|  |  | eCO_2_ | 39 ± 1 | 37 ± 1 |  |  | 37 ± 2 | 36 ± 1 |  |  |
|  | T3 | aCO_2_ | 29 ± 2 | 31 ± 1 | 33 ± 2 | 32 ± 2 | 30 ± 1 | 27 ± 1 | 32 ± 2 | 30 ±1 |
|  |  | eCO_2_ | 33 ± 2 | 35 ± 1 | 30 ± 2 | 32 ± 1 | 26 ± 2 | 29 ± 1 | 30 ± 1 | 27 ± 1 |
| Flag Leaf N_area_  (mmol m^-2^) | T2 | aCO_2_ | 97 ± 20 | 108 ± 4 |  |  | 125 ± 7 | 114 ± 3 |  |  |
|  |  | eCO_2_ | 121 ± 8 | 112 ± 4 |  |  | 113 ± 7 | 109 ± 5 |  |  |
|  | T3 | aCO_2_ | 70 ± 7 | 104 ± 5 | 87 ± 12 | 82 ± 12 | 74 ± 4 | 71 ± 16 | 63 ± 13 | 81 ± 6 |
|  |  | eCO_2_ | 76 ± 6 | 92 ± 5 | 79 ± 11 | 73 ± 6 | 65 ± 8 | 80 ± 5 | 79 ± 3 | 71 ± 5 |
| Leaf N  (mg g^-1^) | T1 | aCO_2_ | 48 ± 2 |  |  |  | 49 ± 1 |  |  |  |
|  |  | eCO_2_ | 53 ± 1 |  |  |  | 53 ± 1 |  |  |  |
|  | T3 | aCO_2_ | 35 ± 1 |  |  | 31 ± 3 | 34 ± 1 |  |  | 33 ± 1 |
|  |  | eCO_2_ | 36 ± 1 |  |  | 36 ± 2 | 28 ± 1 |  |  | 30 ± 1 |
|  | T4 | aCO_2_ | 9.6 ± 1 |  |  | 10 ± 1 | 7.4 ± 0.5 |  |  | 8 ± 0.4 |
|  |  | eCO_2_ | 12 ± 1 |  |  | 11 ± 1 | 7.5 ± 0.7 |  |  | 6 ± 0.1 |
| Stem N  (mg g^-1^) | T1 | aCO_2_ | 31 ± 2 |  |  |  | 39 ± 2 |  |  |  |
|  |  | eCO_2_ | 38 ± 4 |  |  |  | 39 ± 1 |  |  |  |
|  | T3 | aCO_2_ | 11 ± 1 |  |  | 13 ± 1 | 14 ± 3 |  |  | 23 ± 1 |
|  |  | eCO_2_ | 11 ± 1 |  |  | 16 ± 1 | 11 ± 1 |  |  | 16 ± 1 |
|  | T4 | aCO_2_ | 9.7 ± 1.3 |  |  | 6.8 ± 1.6 | 6 ± 1 |  |  | 5.6 ± 2 |
|  |  | eCO_2_ | 10.1 ± 1 |  |  | 11 ± 2 | 3 ± 0.3 |  |  | 6.7 ± 2 |
| Root N  (mg g^-1^) | T1 | aCO_2_ | 12 ± 1 |  |  |  | 15 ± 1 |  |  |  |
|  |  | eCO_2_ | 14 ± 1 |  |  |  | 12 ± 0 |  |  |  |
|  | T3 | aCO_2_ | 15 ± 0 |  |  | 14 ± 0 | 14 ± 1 |  |  | 17 ± 1 |
|  |  | eCO_2_ | 17 ± 2 |  |  | 16 ± 0 | 12 ± 1 |  |  | 11 ± 0 |
|  | T4 | aCO_2_ | 11 ± 1 |  |  | 14 ± 0 | 15 ± 0 |  |  | 13 ± 1 |
|  |  | eCO_2_ | 13 ± 1 |  |  | 14 ± 1 | 10 ± 1 |  |  | 11 ± 1 |


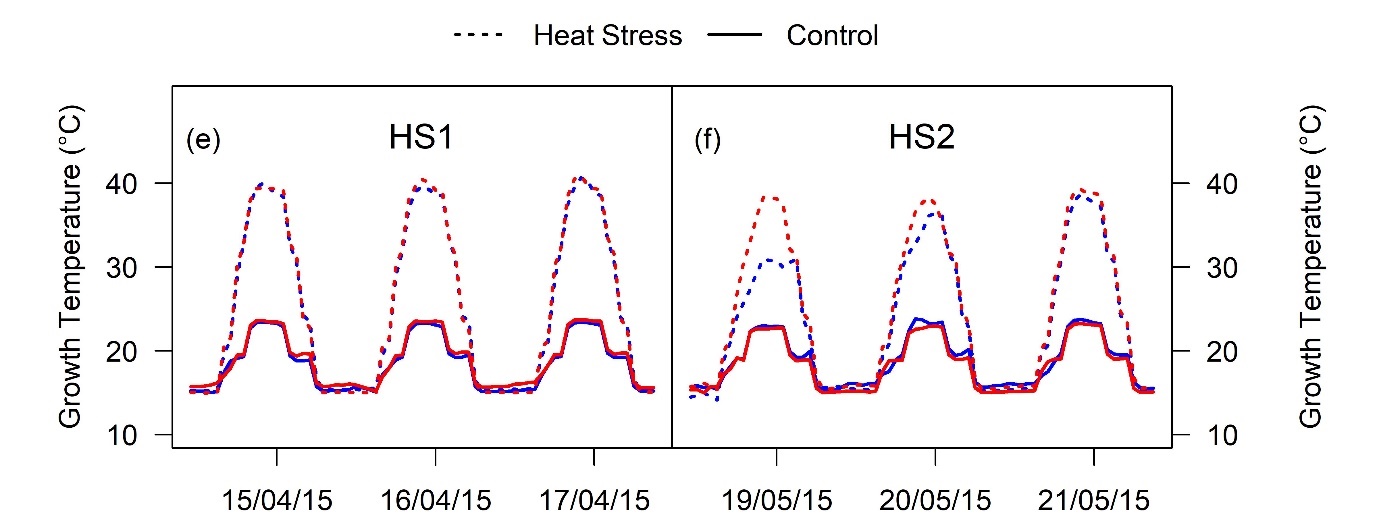

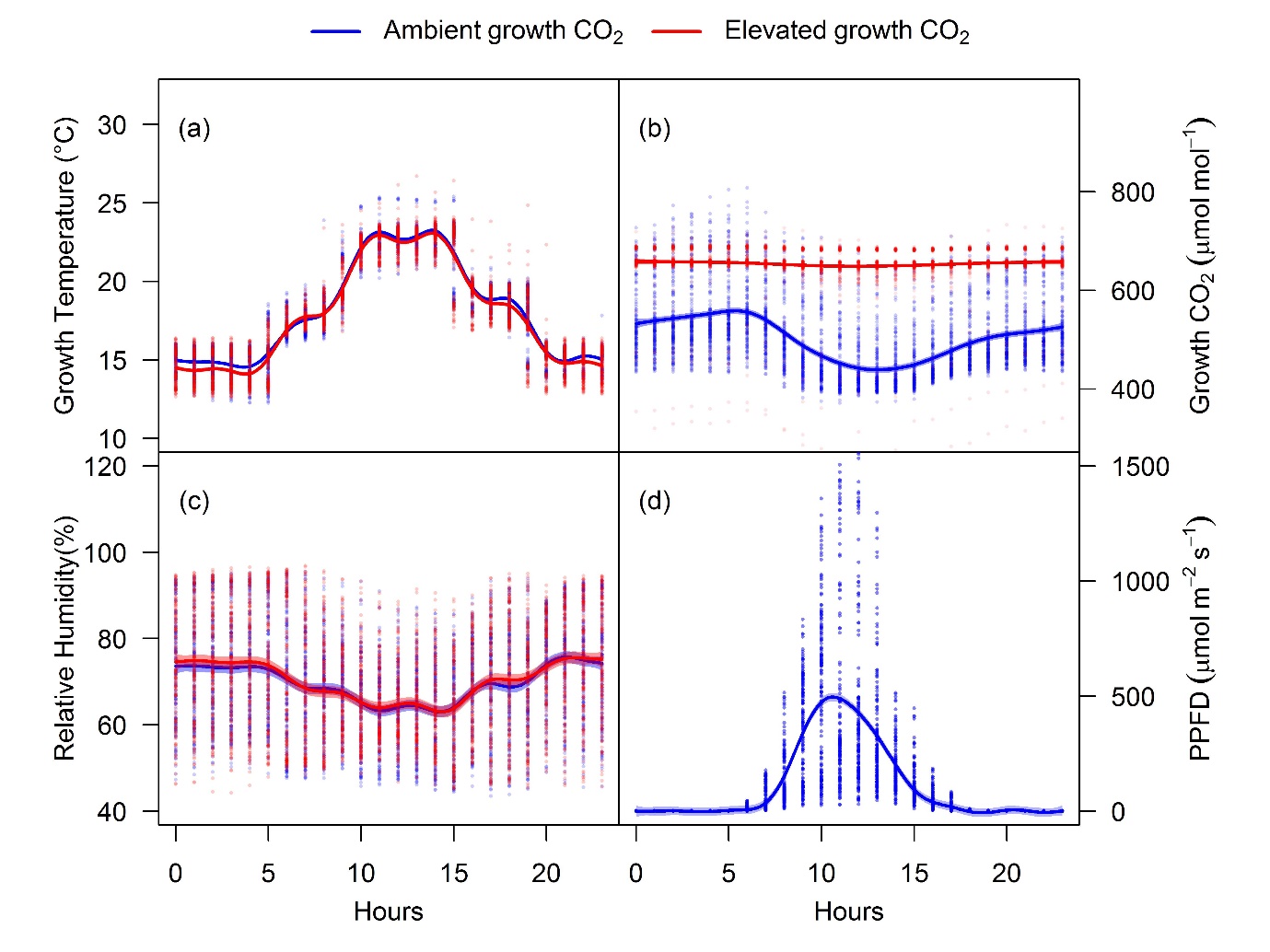
Figure S1. Glasshouse growth conditions. Glasshouse conditions during the experimental growth period; growth temperature (a), growth CO_2_ (b), relative humidity (c) and PPFD (d). In panels a, b, c and d, the solid cultivars represent the growth averages, while the faint data points show all collected values. For HS1 (e) and HS2 (f), the solid cultivars represent control temperature and dotted cultivars represent the heat stress temperature. Ambient and elevated CO_2_ are depicted in blue and red color, respectively.


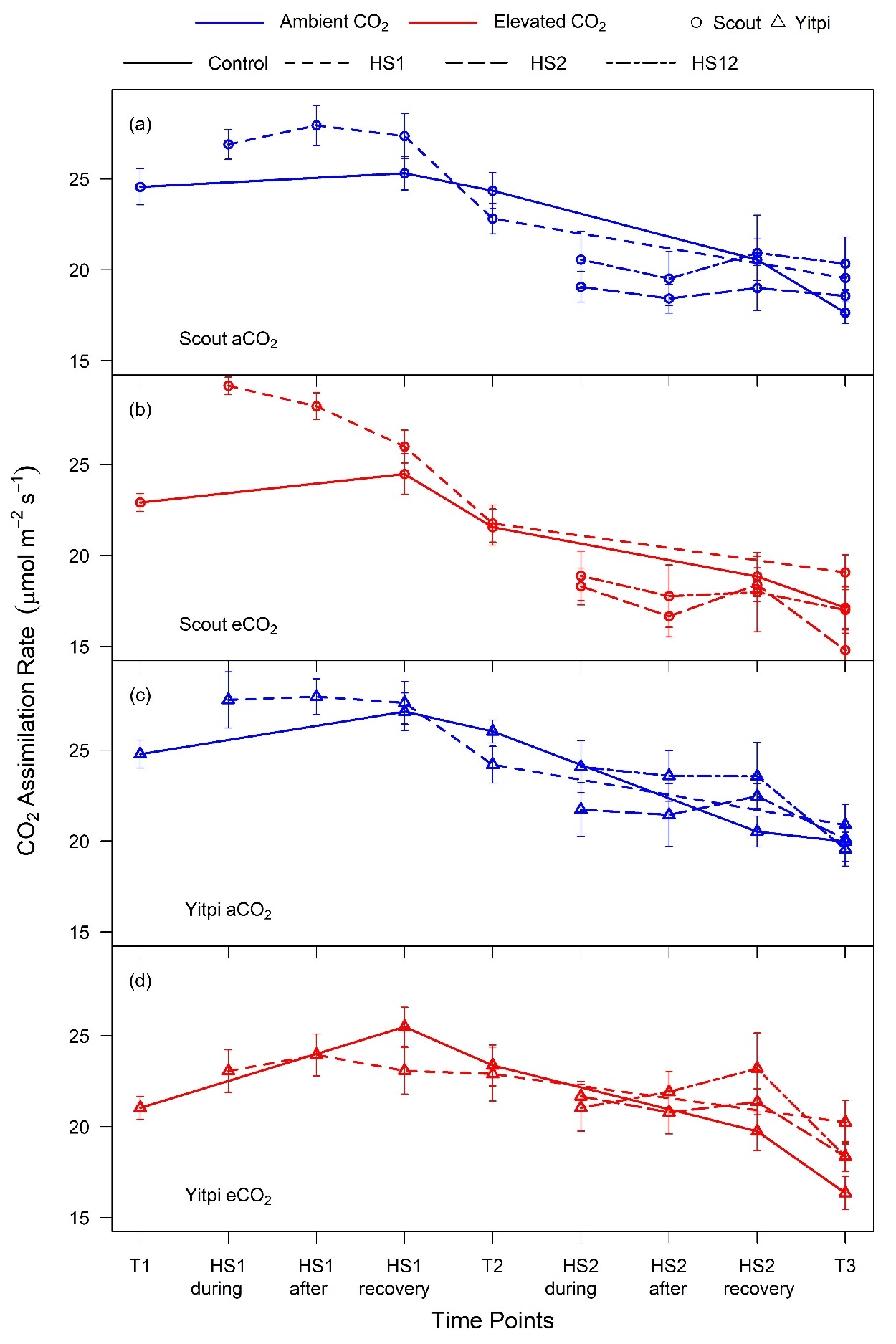


Figure S2. Photosynthetic response to growth at eCO_2_ and heat stresses (HS1 and HS2). CO_2_ assimilation rates measured at common CO_2_ (400 μl L^-1^) and 25°C in aCO_2_ grown Scout (a), eCO_2_ grown Scout (b), aCO_2_ grown Yitpi and eCO_2_ grown Yitpi (d). Ambient and eCO_2_ CO_2_ grown plants are depicted in blue and red, respectively. Measurements were performed during, after, at recovery stage of heat stress and at time points T1, T2 and T3.


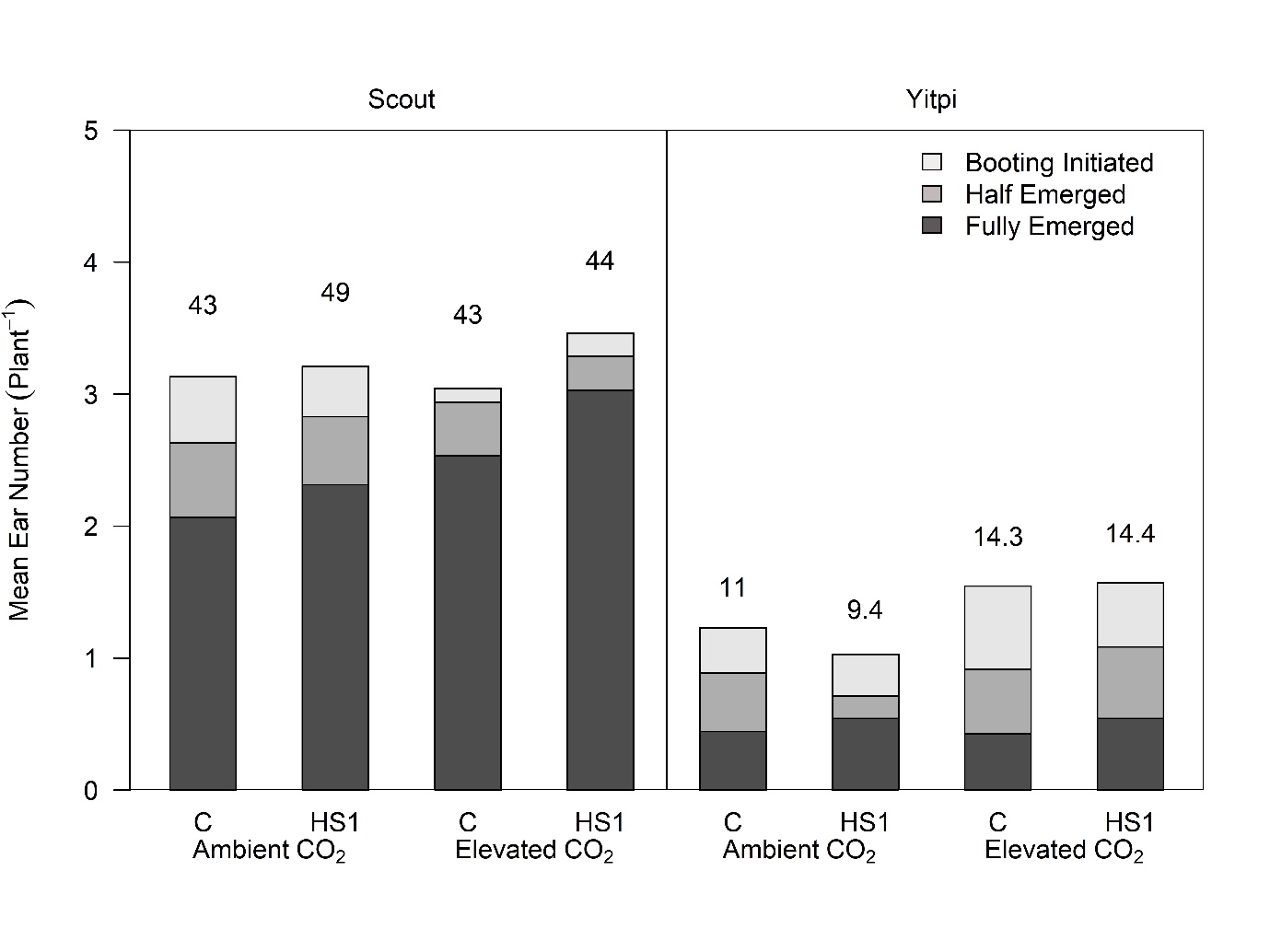


Figure S3. Response of ear development in Scout and Yitpi to eCO_2_ and HS at the booting stage (T2). Data includes plants grown at ambient or elevated CO_2_ and exposed to heat stress 1 (HS1). Numbers above each bar denote the percentage of ears relative to the total number of tillers.


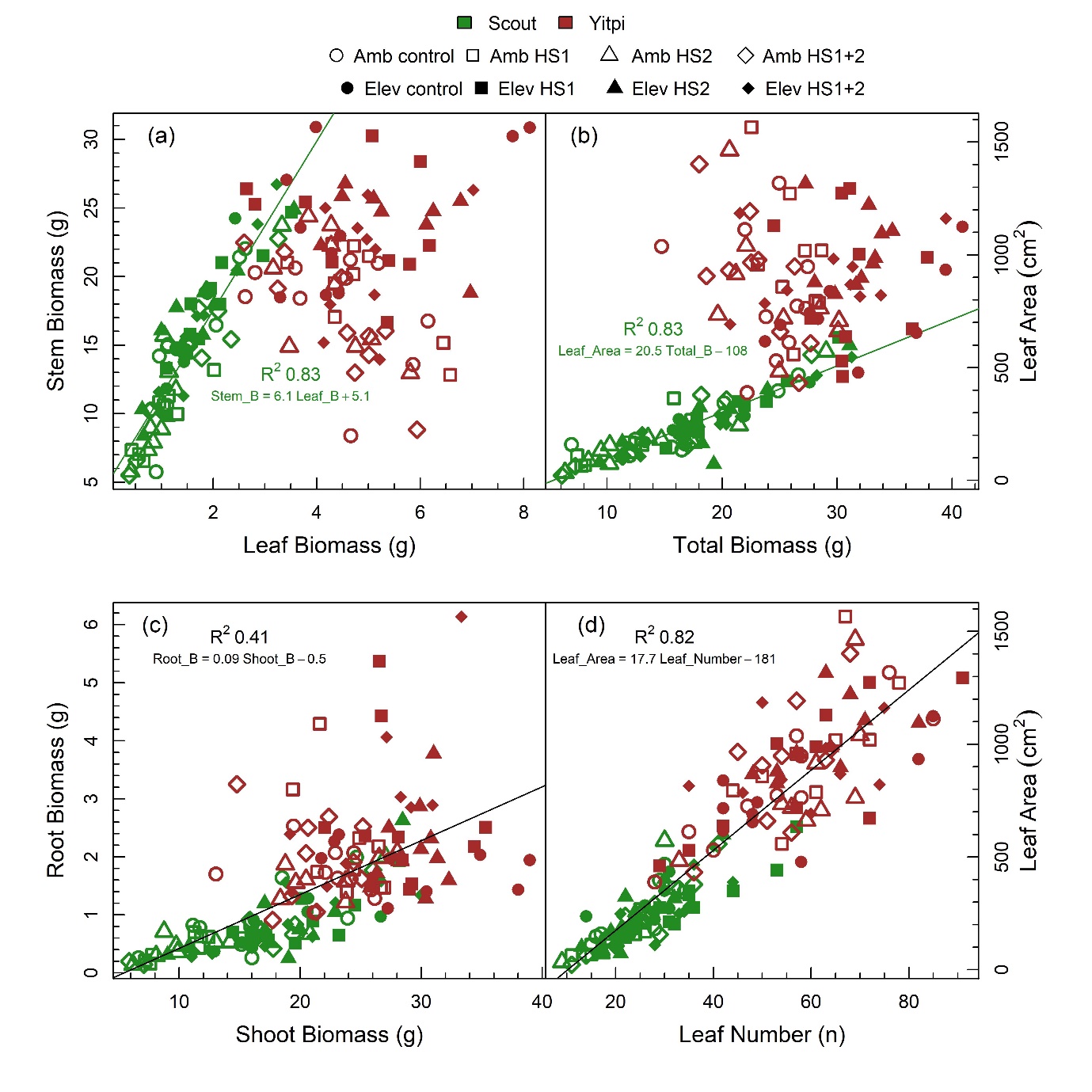


**Figure S4. Relationship between dry mass and morphological parameters measured at anthesis (T3).** Linear regression plotted for relationships between stem dry mass and leaf dry mass (a), total dry mass and leaf area (b), root dry mass and shoot (stem + leaf) dry mass (c) and leaf number and leaf area (d). Scout and Yitpi are depicted using green and brown color respectively. Ambient CO_2_ and eCO_2_ grown plants are depicted with open closed symbols, respectively. Heat stress (HS) levels are depicted in different shapes and include plants not exposed to any heat stress (control), plants exposed to heat stress 1 (HS1), plants exposed to heat stress 2 (HS2) and plants exposed to both the heat stresses (HS1+2).
